# Researches on the Symptoms and Diagnosis of Aneurism of the Thoracic Aorta

**Published:** 1835

**Authors:** 


VI. *Researches on the Symptoms and Diagnosis of Aneurism of the Thoracic Aorta.* By George Green, M. D., Fellow of the Royal College of Physicians, etc., etc.—We are reminded, in this interesting paper, of the difficulty which has existed in detecting aortic thoracic aneurism, in its incipiency—that Laennec was not aware of the utility of the stethescope, in this respect—that without the aid of this instrument this form of aneurism cannot always be distinguished from other tumors—that Bertin and Dr. Hope maintain, that aneurism may be detected in the chest, by means of the stethescope, as certainly as affections of the heart or lungs.

Dr. Green, entertaining views in accordance with those of Bertin and Hope, details several cases in support of the opinion, that the stethescope, by careful and repeated applications, will serve to point out thoracic aneurism, before there are any other evidences of such disease.

Case 1. *Double aneurism of the thoracic aorta. Sudden death from rupture of the sac, into the left bronchial tube.*

Luke Moran, a sawyer, was admitted into the Whitworth Hospital, 7th of April, 1834. For two years he had had a cough, attended with great dyspnœa, and frothy expectoration —the cough paroxysmal, generally at night—there were irregular lancinating pains throughout the chest. Symptoms so severe as to prevent his attention to business the last six months.

At the time of admission, he coughed for half an hour, and felt a sense of impending suffocation. The least exercise increased the dyspnœa; most distress at the top of the sternum. During inspiration the trachea was deeply drawn behind that bone—'breathing laryngeal,' there were darting pains through the chest, slight dysphagia—the radial pulses the same; no œdema or numbness of the upper extremities; the chest sounded clear all over, on percussion, a distinct impulse was given to the stethescope, in the upper third; and, on either side of the sternum, stronger than that of the heart; no distinct bruit; respiration in the left lung feeble; loud and clear in the right, especially on a forcible inspiration. A distinct impulse down the back, on the left of the spine, no purring or tremor present, a distant but distinct bruit de soufflet; sounds of the heart normal. "When the instrument was applied to the axilla, or to the back, in the situation mentioned, the respiration appeared to be almost laryngeal in the top of the left lung, but was natural in the right, and loud and distinct." Five days after admission this patient died suddenly from rupture of the aneurism.

This case, having been advanced before the patient was admitted into the hospital, does not afford any thing directly in support of the point which Dr. Green proposes to establish, viz: that the stethescope may be applied to discover aneurism of the thorax, before it is cognizable by any other means. It does not, therefore, seem necessary to pursue the case further. It may suffice to say, that, as usual in this form of aneurism, the coats of the aorta were found diseased, having "atheromatous deposits" between its coats.

Case 2, Was a woman of 27 years of age, full habit, florid complexion—admitted into the Whitworth Hospital, 8th of January, 1835. About two months before admission she fell down stairs, on her back, after which she felt pain and weakness in that part. The pain is now seated from the second to the fourth dorsal vertebræ. Six weeks before admission she was attacked by a severe cough, a sense of tightness across the chest, and oppression. The cough was loud, harsh, and of a peculiar ringing character; and there was a retraction of the trachea, deep behind the sternum, similar to that noted in case first—there was frothy expectoration somewhat copious. The cough came in violent paroxysms, chiefly at night; threatening suffocation, and obliged her sometimes to sit up; some difficulty in swallowing, which is referred to the upper third of the sternum, where the morsel appears to stop, and requires the aid of drink to convey it down. There is pain on pressure of the third and fourth spinous process of the dorsal vertebræ. The pain is of a shooting character, shooting across from the spine to the sternum, and completely traversing the thorax. Pulse in both wrists the same as to force and frequency. No numbness, or œdema, of the upper extremities; respiration hurried.

Percussion afforded a clear sound all over the chest; respiration in the right distinct, but attended by a blowing sound, when the instrument was applied posteriorly, about the situation of the bronchial tube, or in the axilla of the right arm. Respiration in the left lung was feeble and sometimes nearly inaudible. An obscure impulse, accompanied by a bruit de soufflet in the interscapular region. Both of these phenomena most distinct at the left side of the spine, and confined to the part where she felt pain from pressure, most remarkable when the arterial system was excited. Anteriorly an impulse was communicated to the stethescope at the sterno clavicular articulation, and sub-clavian region of the left side. The impulse was attended with a double sound, and was somewhat stronger than that of the heart. An obscure bruit de soufflet was heard in the same situation. The impulse and sounds of the heart were natural. She complained of pain upon pressing the top of the sternum. The symptoms were occasionally moderated by general and local bleeding.

Two days before the patient's death, and twenty-sixth after her admission, the following note was made. There is a strong double impulse a little below the sterno-clavicular junction; at the left side of the medium line of the sternum, accompanied by two distinct sounds, and a clear bruit de soufflet. Impulses diminished towards the heart, which retains its natural impulse and sounds. No expansive pulsation on pressing the fingers deep behind the clavicles; left jugular vein turgid. The stethescope detected a bruit de rape, this diminishes as the instrument is carried down on the left side of the spine. There was now to be seen an elevation by looking along the anterior surface of the chest, at the top of the sternum. Here, too, pulsation can be felt, and percussion produces pain. The dyspnœa caused great alarm. Examination after death disclosed the rupture of an aneurismal tumor of the aorta. Another aneurismal tumor was found in a state of moderate development.

We have reason to believe that in this case the stethescope aided, in some degree, to ascertain the nature of the disease in its early stages; but the case is not calculated to be useful, so much on this account, as in regard to its serving to awaken closer attention to the stethescope, as a means of discovering aneurism, when concealed in this deep and hidden situation.

Case 3. This case having been detected whilst the aneurism must, as Dr. Green supposes, have been in a very incipient state, is on that account important. Michael Hughes, a butler, aged 38, a stout, well made man, was admitted into the Meath Hospital, complaining of great dyspnœa, cough, and wandering pains about the back and chest. Chest sounds clear throughout on percussion. Respiration in the right lung intensely peurile, altogether absent in the superior portion of the left, and only audible in the interior portion. The patient drawing in a deep breath while the stethescope is applied to the left axillary region, no sound is heard during the first half of the inspiration, but, during the latter half, the air appears to overcome some obstruction to its entrance, and suddenly rushes in to expand the lungs. The left side of the chest is almost immovable during respiration, but the right acts freely. On applying the hand to the upper part of the left side, no vibration of the voice could be detected. The vibration is very feeble on the same side, but well marked on the right side; impulse and sound of the heart natural; a strong double pulsation is heard over the sub-clavicular region of the side, also over the postero-superior portion of the same side, and on applying the hand over the former situation distinct impulse is communicated to it. No bruit de soufflet could be detected. The cough is hard and ringing, and of a peculiar croupy character; expectoration scanty, consisting of frothy mucus; respiration hurried. Pulse 86, and moderately full; tongue clean, appetite good; never had any difficulty in swallowing, or pain in the throat. He attributes his complaint to cold caught five months ago from sleeping in a damp pantry, shortly after which he became affected with perspirations, wandering pains in the back and sides, increased upon exertion, and attended with some dyspnœa. The pain in the back he describes as being of a lancing nature, resembling that which would be inflicted by a knife. The difficulty of breathing increased, with inability to use any violent exertion, and after some time was attended by a loud dry cough.

This patient was afterwards examined at the Richmond Hospital, by Dr. McDowell, at which time there was a striking sameness of symptoms—a gradual increase of severity in the symptoms took place till the patient died suddenly during a fit of coughing, throwing up a good deal of blood just before death.

Dissection disclosed the bursting of an aneurismal tumor within the thorax; and, as usual, the arterial coats were found diseased. Up to the death of this patient the chest sounded clear on percussion, showing, of course, that no aid is to be derived from this expedient in cases of aneurism.

Our examination of the foregoing cases leads us to the conclusion that we cannot abridge Dr. Green's reflections upon these, without lessening their importance. However little we may gain, in a practical point of view, by the more intimate knowledge of the signs of thoracic aneurism in its incipiency, still it is desirable that we be able to detect this disease early. A proper prognosis may enable us to advise palliative measures, at least, and often with the consolation that our knowledge serves to fix our attention, and enables us to advise on that correct knowledge. Moreover, nature having now and then conducted these affections to a favorable termination, leaves a slight hope that, by a well directed course of conduct on the part of patients, and correct advice of the physician, they may be treated with hope of prolonging life, and a belief of possible restoration.

A strict avoidance of all exciting causes, whether it be in regard to exercise, food, mental quietude, occasional depletion, etc., may sometimes, no doubt, be of great advantage to persons thus unfortunately situated. Nor should the plan of abstinence, and other depletory means, be lost sight of, even with the hope of curing in the early stage of the disease—we mean the plan, or something similar to it, of Valsalva.

H. G. J.

The result then of these cases, together with those already published, particularly by Dr. Stokes* and Bertin,† proves that we are in possession of a number of physical signs, which, when attentively studied in connection with the general symptoms, will lead us to a knowledge of this obscure disease, even before it has made any considerable progress. The necessity of taking every phenomenon into consideration, is obvious. We should recollect that our diagnosis ought never to be founded on one or two, but on a series of observations, carefully noted and compared with each other.

I have before mentioned that there is a portion of the aorta where some of those signs must necessarily be absent. As far as the different portions of the arch of the aorta are concerned, the diagnosis of aneurism of the vessel may be said to be in a great measure rescued from the obscurity in which it was formerly involved, and for which we are much indebted, amongst others, to the observations of Dr. Stokes.—But when we come to consider the signs and symptoms we are able to collect when it occurs in the remainder of its course in the thorax, we

*Dublin Medical and Surgical Journal, vol. v.

†Observations 37 and 38, Maladies de Cœur.

find that they are diminished in number, and are often so feebly characterized, as to escape our notice. It may be superfluous, perhaps, to give an example in proof of this assertion, when so many are already on record. The following case, however, which occurred at the Whitworth Hospital, is so much in point that I will here mention it. The case was taken by Mr. Mayne, who was clinical clerk at the time the man entered the hospital. I had not myself an opportunity of examining the patient. I shall give a very condensed account of it from the notes furnished to me by Mr. Mayne.

Laurence Duffy, aged 43, admitted 12th July, 1833, states he had been healthy all his life, until about nine months previous to his admission, when he began to experience a difficulty in swallowing, the increase of which induced him to apply for assistance. When questioned, he complained of difficulty in swallowing, which he referred to the centre of the sternum, where the effort was attended with pain followed by vomiting. Fluids passed down without difficulty; his bowels were constipated; the chest sounded well throughout; the respiration over its entire extent was perfectly natural. His bowels were freed by a calomel bolus, and purgative mixture. A slight cough, which he had on his entrance, altogether disappeared for seven days after his admission, and no symptom whatever was complained of, but the dysphagia. He died suddenly on the night of the seventh day from his admission.

On dissection, an aneurism was found corresponding to the fifth and sixth dorsal vertebræ, about the size of an orange, and it had burst into the right pleura. The tumor had slightly bent the œsophagus, which formed a curve, the concavity towards the sac.

Here then is an instance where, if we were to rely on the general signs, we should have had but one to excite our suspicions of the disease; and this should lead us in all cases of long-continued dysphagia, to institute an accurate examination for the physical signs of aneurism. A question then naturally arises: what are we to expect from such an examination, when aneurism exists in this situation? It will be obvious that when the mouth of the sac is situated below the fourth dorsal vertebræ, we cannot expect to find the same modifications of the respiration as those above mentioned. I mean this remark to apply to the incipient stage of the disease, for when it is considerably advanced the expansion of the sac upwards may of course produce pressure on either of the bronchial tubes, but even such an occurrence could not happen for a considerable length of time, when the tumor is situated opposite the eigthh or tenth dorsal vertebræ. It might perhaps be important to observe in such a case, whether the patient complained of pain on deep inspiration at the postero-inferior portion of the lung, or whether pains resembling those of pleurisy exist about this situation, for if the sac should contract adhesions to the pleura, it might not be unreasonable to expect pains similar to those that precede the advance of tubercles to the surface of the lung. When the sac has so far increased as to produce an absence of respiration in any considerable portion of the lung, the nature of the case will be, in all probability, apparent from other symptoms.

On reflecting upon the symptoms which would attend an aneurism so low down in the thorax, I at first imagined, that if the tumor produced an impediment to the aortal circulation, we might be able to ascertain it by the feeble impulse of the abdominal aorta, when the stethescope was pressed firmly down upon it, some way above the umbilicus, but on considering the cases that have been published of obliteration or contraction of this vessel, I am not inclined to suppose that this phenomenon would exist. Many cases of such a change in the artery, as I have mentioned, are now on record. The latest published is in an interesting communication from Mr. Nixon, in the fifth volume of the Dublin Medical Journal, where copious references are made to those previously noticed. From examining the plates which illustrate these contractions of the vessel, it will be seen that the aorta immediately below the constriction is abundantly supplied by collateral vessels, and no perceptible diminution of the calibre of the tube can be recognized. It is remarkable that this phenomenon has occurred in so many cases, near the situation of the ductus arteriosus, and it may become a question for further consideration, whether the process established by nature for the closure of that tube at birth, may not be extended to the aorta in its vicinity, and thus constitute either a congenital malformation, or establish a predisposition to aneurism in this part of the vessel.

The collateral circulation, I have alluded to, is attended with such an enlargement of the vessels that carry it on, that I was in hopes we might be led to infer the existence of the stricture, by attending to the increased pulsations in these arteries; but, unfortunately, they are so numerous and deeply seated, as to be without the reach of any means we can apply to discover their pulsations. In all probability, also, should the closure occur from the pressure of an aneurism, these changes in the collateral vessels will be a gradual process, so that no sudden diminution of the pulsation of the vessel could be discovered below the stricture. The principal alterations that occur in these cases, are in the intercostal arteries, and their anastomosis with the mammary, etc. as is well seen in a plate in Meckel's Archives, 1827. The case of constriction, published by Meckel, occurred about the situation of the ductus arteriosus. It was accidentally discovered in the body of a patient, who died at Bonn, but of whose previous history nothing could be ascertained. In the plate which illustrates this lesion, and for the inspection of which I am indebted to Dr. Graves, who directed my attention to the case, these arteries are seen remarkably developed and tortuous, but as I have just observed, they would be too deep from the surface to enable us to recognize their pulsations during life.

In concluding these remarks, I would wish to direct attention to the obscurity which still attends the diagnosis of aneurism of the inferior descending portion of the aorta, an obscurity which does not attach in so great a degree to aneurisms of the different portions of the arch. Since I have entered on the investigation of this subject, I have not been able to examine a case of the disease in this situation, but it is not improbable that those who have, will be enabled by their observations to elucidate this interesting point of pathology.

As far as I am at present enabled to offer an opinion, the principal symptoms and physical signs on which I would be inclined to diagnose the existence of an aneurism in the situation I have just mentioned, would be as follows:

1st. An impulse limited in extent, and decreasing in intensity as the stethescope is applied above or below the situation of the suspected tumor. This sign is more valuable, if it occurs on the right side of the spinal column, which is not the situation, anatomically speaking, where an impulse should be perceived.

2d. A bruit de soufflet or a bruit de rape, heard in the suspected tumor, and not observable in any other considerable portion of the aorta or in the heart. It is necessary in every instance to examine the heart, for within these few days I have met with a casé in the Whitworth Hospital, where a loud bruit de rape accompanies the second sound of the heart, this bruit is heard down the spine, along the course of the aorta. It is heard also in the carotid and axillary arteries, even to the elbow-joint. I have satisfied myself that in this case a double sound is heard in these arteries. The name of the individual in whom these phenomena occur, is William Connor, and several of the class of the Whitworth Hospital have heard both the bruit and the sound in the situations mentioned.

3d. The production of pain on pressing the vertebral column over the site of the impulse.

4th. The production of pain referred to a portion of the lung near the situation of the impulse, on a forcible inspiration.

5th. Dysphagia referred to some point below the middle third of the sternum, or to a point nearly opposite the impulse.

Lastly, it may be worth while to contrast the pulsations of the abdominal aorta in the epigastrium, with other arteries and with the impulse of the heart.

We should take into consideration, also, (provided the above signs are well marked,) the evidence we may obtain with respect to the position of the tumor from the absence of some symptoms, such, for instance, as the non-existence of a laryngeal croupy cough; difference of pulsation in the radial arteries; or numbness or œdema of the upper extremities.

I regret that the time afforded for getting this paper ready for the present number of the Journal, is not sufficient to enable me to consider this subject more at large. As, however, I have had no opportunity of studying the phenomena attendant on an aneurism in the part of the thoracic aorta, to which I wish to draw attention, I feel it more prudent to reserve any further observations for a future opportunity. I am sensible of having left much concerning this interesting subject as yet untouched, but in giving the cases above detailed, I shall be satisfied if they tend to strengthen our confidence in the diagnosis we are able to arrive at, by the additional aid of auscultation. The publication of such cases is the more necessary, from considering the doubtful terms in which Laennec expressed himself concerning the value of the stethescope, as applied to the diagnosis of the disease in question. We should, however, recollect, that the obscurity which the brilliancy of one mind is unable to dissipate, may be cleared up by the labors of many, and that the contributions of individuals for this purpose are, in the language of Abernethy, "like lights shining from many quarters." pp. 413–417.

